# Prepartum Blood Biochemistry and Periparturient Disease Risk in Dairy Cows: A Retrospective Two-Farm Study

**DOI:** 10.3390/ani16182946

**Published:** 2026-09-19

**Authors:** Woojae Choi, Younghye Ro, Eunhui Choe, Danil Kim

**Affiliations:** 1 Department of Theriogenology, College of Veterinary Medicine, Chungbuk National University, Cheongju 28644, Republic of Korea; wjchoi@cbnu.ac.kr; 2 Department of Large Animal Medicine, College of Veterinary Medicine, Kangwon National University, Chuncheon 24341, Republic of Korea; dura82@kangwon.ac.kr; 3 Farm Animal Clinical Training and Research Center, Institutes of Green-Bio Science and Technology, Seoul National University, Pyeongchang 25354, Republic of Korea; choieh08@snu.ac.kr; 4 Department of Farm Animal Medicine, College of Veterinary Medicine, Seoul National University, Seoul 08826, Republic of Korea; 5 Research Institute for Veterinary Science, Seoul National University, Seoul 08826, Republic of Korea

**Keywords:** transition period, dairy cattle, retained placenta, ketosis, milk fever, biomarker

## Abstract

Periparturient diseases are a major cause of economic loss and impaired welfare in dairy cattle. We screened 29 prepartum biochemistry and hematology parameters for association with retained placenta (RP), dystocia, abortion/stillbirth, ketosis, and milk fever in 419 calving events from two Korean dairy farms (DF1, *n* = 248; DF2, *n* = 171). Each calving was sampled twice prepartum on alternating biweekly schedules, resolved into an early (week −4/−3) and late (week −2/−1) window. Parameters were compared between affected and unaffected calvings using linear models adjusted for parity, body condition score (BCS), and farm, with Benjamini–Hochberg false discovery rate (FDR) correction, and were then entered into multivariable logistic models. After adjustment and FDR correction, two markers remain associated with disease: lower prepartum calcium for milk fever and elevated direct bilirubin with reduced cholesterol and triglycerides for ketosis, both detectable in both windows three to four weeks before calving. Multivariable models discriminated milk fever (AUC = 0.81) and ketosis (AUC = 0.75), with comparable discrimination when models were applied to the other farm. A prepartum bilirubin signal for RP, consistent with an emerging bilirubin–RP association reported via plasma metabolomics, was detectable but did not survive confounder adjustment and is reported as hypothesis-generating. Using only routine clinical biochemistry and a practical biweekly schedule, these parameters were associated with subsequent milk fever and ketosis weeks before calving, identifying them as candidate markers that require prospective validation.

## Simple Summary

In the weeks around giving birth, dairy cows are especially likely to fall ill, which harms their health and causes economic losses for farmers. Catching cows at risk before they become sick would allow earlier care, but reliable early warning signs are hard to find. We studied routine blood samples taken from cows on two farms in the month before calving to see whether they could identify cows that would later develop common calving-related disorders. We found that two simple blood measures—one reflecting calcium levels and one reflecting how well the liver is working—identified cows at higher risk of low blood calcium and of a common energy-related disorder, three to four weeks before birth. Because these measures come from ordinary blood tests that most veterinary laboratories already run, they could offer farmers and veterinarians an affordable way to identify vulnerable cows early, take preventive action, and improve both animal welfare and farm productivity.

## 1. Introduction

The periparturient (transition) period, spanning approximately four weeks before to three weeks after calving, is characterized by profound metabolic and endocrine changes that predispose dairy cows to a cluster of interrelated diseases, including retained placenta (RP), dystocia, abortion/stillbirth, ketosis, and milk fever [1,2]. As parturition approaches, declining feed intake combines with the escalating energy demands of late gestation and the onset of lactation to produce a state of negative energy balance, characterized biochemically by mobilization of adipose tissue (elevated non-esterified fatty acids, NEFAs), incomplete hepatic oxidation of fatty acids (elevated beta-hydroxybutyrate, b-HB), and reduced circulating glucose and cholesterol [3,4]. In parallel, the abrupt onset of colostrogenesis imposes a sudden calcium demand that, in cows with insufficient compensatory intestinal absorption or bone mobilization, produces milk fever [5]. These metabolic and mineral disturbances do not occur in isolation: negative energy balance impairs neutrophil function and uterine involution, increasing the risk of RP and metritis [6,7,8], while milk fever reduces smooth muscle contractility, predisposing to dystocia and further RP [9,10]. This physiological interdependence motivates a multi-parameter, multi-disease approach rather than single-marker, single-disease screening.

These disorders cause direct economic losses through treatment costs, reduced milk yield, and involuntary culling, and increase susceptibility to secondary disease through this shared pathophysiology [11]. Consequently, considerable effort has been devoted to identifying prepartum blood biomarkers capable of predicting disease susceptibility before clinical disease onset [12,13], so that preventive interventions (e.g., anionic diet supplementation, monensin boluses, and closer monitoring) can be targeted to cows most likely to benefit [14,15].

Elevated NEFA and b-HB, together with reduced total cholesterol, are well-established predictors of ketosis [16,17], while low prepartum blood calcium has been linked to postpartum subclinical milk fever [18,19]. However, most of these studies rely on a single prepartum sampling time point (typically 1–3 weeks before calving) and data from a single farm, limiting both the temporal resolution needed to identify the optimal screening window and the generalizability of reported thresholds across management systems and parity structures. Furthermore, while classical RP predictors emphasize NEFA, body condition score (BCS), b-HB, aspartate aminotransferase (AST), and γ-glutamyl transferase (GGT) [20], a bilirubin-RP association has recently emerged from untargeted plasma metabolomics [21,22]. Although sporadic alterations in total bilirubin have been noted in broad metabolic profiles [21,23], the application of this biomarker within a standardized screening framework has not been extensively explored. Therefore, this study aimed to investigate the potential of bilirubin as a practical, herd-level screening tool for RP, utilizing routine biochemistry panels that most veterinary diagnostic laboratories can readily perform for large-scale monitoring. Dystocia, abortion, and stillbirth have received comparatively little attention as outcomes of prepartum biomarker screening and were therefore included alongside these conditions.

This study analyzed retrospective prepartum blood biochemistry and hematology from two Korean dairy farms with contrasting parity structures. Building on reports that prepartum metabolic disturbance precedes transition disease, we asked which prepartum parameters—and in which prepartum window—remain associated with RP, dystocia, abortion/stillbirth, ketosis, or milk fever once parity, body condition, and multiple testing are accounted for [24], whether these associations are consistent when the two farms are analyzed separately, and whether the surviving markers are detectable early enough for practical use. Rather than compiling an exhaustive association catalog, we aimed to define a compact, deployable set of prepartum markers, obtainable from a routine biochemistry panel and validated in calving-event-level multivariable models adjusted for parity, BCS, and farm.

## 2. Materials and Methods

### 2.1. Animals, Farms, and Clinical Management Subsection

Retrospective periparturient blood biochemistry and hematology records were obtained from two Holstein dairy farms in Korea (designated DF1 and DF2), both located in the Pyeongchang highland dairy region and operating under different housing systems (DF1, loose housing; DF2, free-stall), with records spanning a 6-year period. The analytic unit was defined as an individual calving event. This yielded 248 calving events (158 cows) for DF1 and 171 calving events (115 cows) for DF2 (Table 1). DF2 cows were predominantly multiparous (parity 2.41 ± 1.45), whereas DF1 cows were predominantly primiparous (parity 1.74 ± 0.97).

Both farms are enrolled in a routine herd-health program operated by the Farm Animal Clinical Teaching Hospital, Seoul National University. Under this program, the attending veterinarians visit each farm biweekly, alternating between transition-cow work and reproductive examination, so that every cow in the transition period undergoes blood sampling for metabolic assessment once every two weeks. The results were used to assess metabolic status before and after calving and to identify animals requiring closer monitoring afterwards. The program operated independently of the present analysis, which was initiated subsequently using these pre-existing records. Blood was collected from the coccygeal vein into K2-EDTA and sodium-heparin tubes and immediately placed in coolers for transport to the laboratory. At each prepartum visit, fetal viability, uterine status, and body condition score (BCS) were confirmed concurrently with sampling. Cows also received a monthly prepartum injection of a vitamin A/D3/E compound (Vigantol-E, Elanco Animal Health Inc., Greenfield, IN, USA) as part of routine herd management, a standard husbandry practice applied independently of this study.

### 2.2. Laboratory Analysis

Whole blood collected in K2-EDTA tubes was used directly for complete blood count (CBC) analysis on a Hemavet analyzer (Drew Scientific, Plantation, FL, USA). The remaining K2-EDTA blood and the sodium-heparin blood were centrifuged at 3000× *g* for 15 min, and plasma biochemistry parameters were measured on a BS-400 chemistry analyzer (Mindray, Shenzhen, China).

### 2.3. Study Design, Sampling Windows, and Outcome Definitions

Every one of the 419 calvings had exactly two prepartum blood draws, following one of two alternating biweekly schedules: weeks −4, −2 (*n* = 184) or weeks −3, −1 (*n* = 208), with a small remainder (*n* = 27) sampled at −4, −1 or −3, −2. We defined two composite prepartum windows for window-resolved analyses: an early window (the −4 or −3 sample, whichever was available for a given calving) and a late window (the −2 or −1 sample); every calving contributed exactly one measurement to each window, so window-level comparisons use the full analytic sample (*n* = 419, less missing values). Twenty-nine biochemistry and complete blood count parameters were measured per sample, spanning leukogram, erythrogram, protein, energy/lipid metabolism, liver function, renal function, and mineral panels. Retained placenta, dystocia, abortion/stillbirth, ketosis, and milk fever were recorded as binary postpartum outcomes. Ketosis and milk fever were defined by postpartum blood biochemistry: ketosis as a plasma b-HB concentration above 1.4 mmol/L, and milk fever as a total calcium concentration of 8.0 mg/dL or below. As used here, “milk fever” therefore denotes biochemically defined hypocalcemia spanning subclinical and clinical cases, rather than clinical hypocalcemia alone. The remaining outcomes (retained placenta, dystocia, abortion/stillbirth) were recorded from clinical calving records. Twinning was excluded from all analyses because of near-absent case numbers (DF1, *n* = 0; DF2, *n* = 5).

### 2.4. Statistical Analysis

For each farm and prepartum window (early, late; defined above), descriptive statistics and normality (Shapiro–Wilk test) were assessed. As the primary screen, each parameter was compared between affected and unaffected calvings using linear models adjusted for parity, BCS, and farm (farm included as a covariate in pooled analyses); the corresponding unadjusted comparison (Welch’s *t*-test if both groups were approximately normal with *n* ≥ 3, or the Mann–Whitney U test otherwise) is reported as a reference (Supplementary Figure S3). To control for multiple comparisons within each disease (2 windows × 29 parameters = 58 tests per family), Benjamini–Hochberg false discovery rate (FDR) correction was applied [25]; *q* < 0.05 was considered significant. Consistency across farms was assessed by requiring a consistent direction of effect in both farms analyzed separately (Supplementary Figures S1 and S2).

For multivariable prediction, RP, ketosis, and milk fever were modeled using calving-event-level data (prepartum parameters averaged across weeks −4 to −1); dystocia and abortion/stillbirth were excluded due to insufficient events. Candidate predictors (univariate *p* < 0.20, collinearity-filtered at r > 0.8) were added to parity/BCS/farm-adjusted models via forward stepwise selection minimizing AIC [26] (entry *p* < 0.15; max 4 predictors). Discrimination was summarized using AUC and McFadden’s pseudo-R^2^. The statistical analyses were performed using Python 3.12 (Python Software Foundation, Wilmington, DE, USA), utilizing the SciPy 1.17 (SciPy Community, Austin, TX, USA) and Statsmodels 0.15 (Statsmodels Developers, Chapel Hill, NC, USA) packages. Five-fold cross-validation was repeated with five random partitions, and the mean AUC is reported.

Because 97 of 273 cows (36%) contributed more than one calving, all final models were additionally re-estimated using generalized estimating equations with an exchangeable working correlation clustered by animal. External validity was assessed by cross-farm validation, training on one farm and testing on the other.

### 2.5. GenAI Use Disclosure

Google Gemini (Google LLC, Mountain View, CA, USA) was used for limited assistance during the figure preparation and statistical analysis processes. All AI-generated outputs were independently checked against the final raw data and thoroughly reviewed by the authors.

## 3. Results

### 3.1. Study Population and Disease Incidence

A total of 419 calving events (DF1, *n* = 248 from 158 cows; DF2, *n* = 171 from 115 cows) met inclusion criteria. DF1 cows were predominantly primiparous (parity 1.74 ± 0.97), whereas DF2 cows were predominantly multiparous (parity 2.41 ± 1.45), while mean prepartum body condition score (BCS) was almost identical (DF1 3.37 ± 0.15; DF2 3.36 ± 0.17). Disease incidence by farm is summarized in Table 1. Retained placenta (RP) incidence was similar between farms (DF1 12.5% and DF2 14.0%), whereas ketosis was more frequent in DF1 (25.4% vs. 16.4%) and milk fever in DF2 (21.6% vs. 12.9%). Dystocia (2.0–3.5%) and abortion/stillbirth (1.6–8.2%) were comparatively rare, and twinning was excluded entirely (DF1 *n* = 0; DF2 *n* = 5).

### 3.2. Prepartum Parameters Associated with Each Disease After Confounder Adjustment

**Figure 1:**
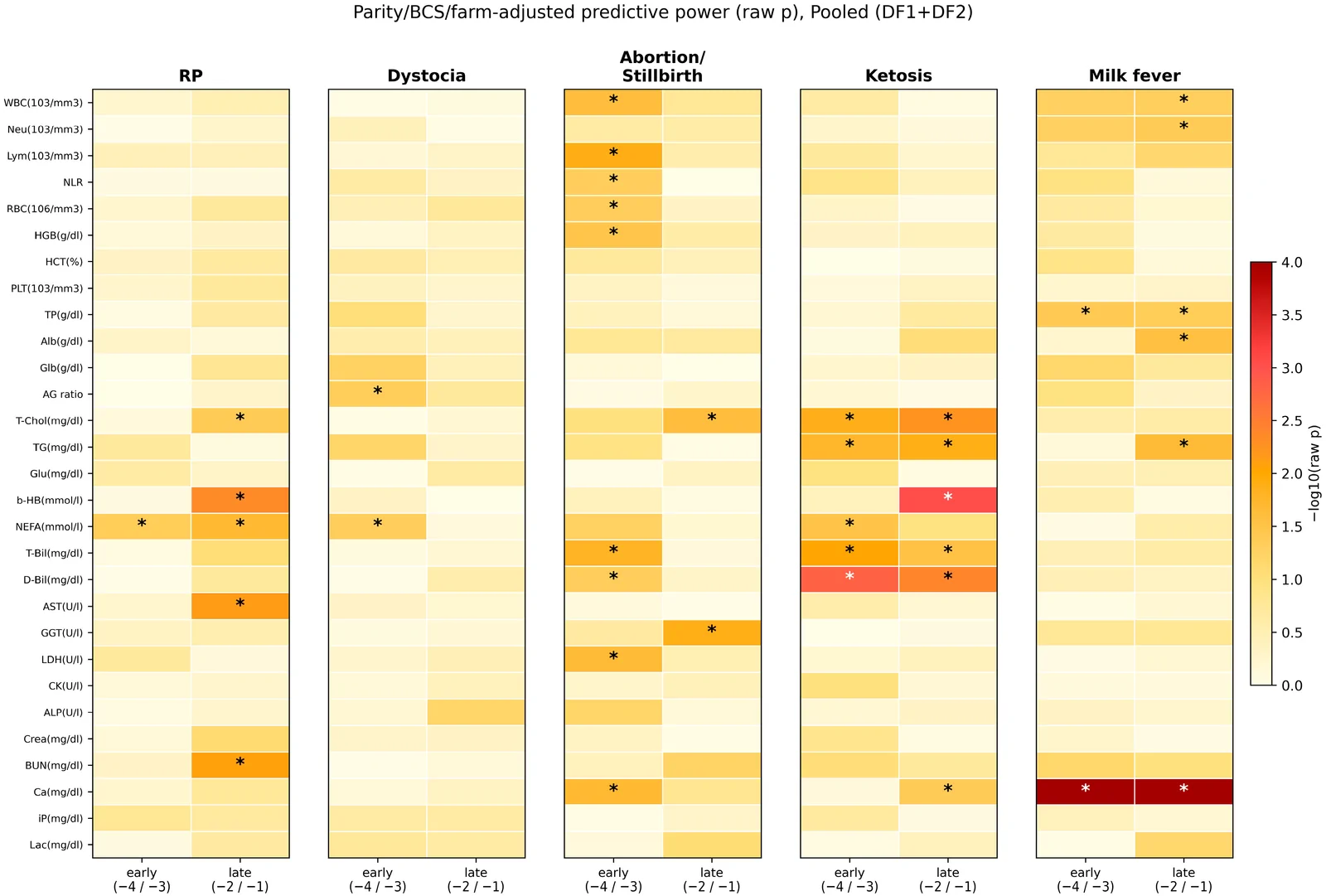
Prepartum parameters associated with each disease before multiple-testing correction (pooled, parity/BCS/farm-adjusted). Heatmap of the association between each of 29 blood parameters and the five diseases, compared between affected and unaffected calvings using linear models adjusted for parity, BCS, and farm. Color encodes the uncorrected −log_10_ (raw p), with stronger associations. Each disease is shown for the early (week −4/−3) and late (week −2/−1) prepartum windows; asterisks (*) mark raw *p* < 0.05.

To identify prepartum parameters associated with each disease while ensuring that any selected association was not merely a reflection of herd parity structure or body condition, every parameter was compared between affected and unaffected calvings in each prepartum window using linear models adjusted for parity, BCS, and farm, and the resulting 58 *p*-values per disease (2 windows × 29 parameters) were corrected within each disease by the Benjamini–Hochberg FDR. The adjusted raw-*p* landscape (Figure 1) retained coherent prepartum signals for four of the five outcomes. Ketosis showed the most extensive independent signature—lower prepartum total cholesterol (T-Chol) and triglycerides (TG) with higher direct bilirubin (D-Bil), total bilirubin (T-Bil), b-HB, and NEFA, concentrated in the late window. Milk fever was independently marked by lower prepartum calcium (Ca) in both windows, with lower total protein and albumin. Abortion/stillbirth retained lower early-window lymphocytes with higher lactate dehydrogenase (LDH) and bilirubin, and lower late-window T-Chol and GGT. RP retained higher late-window b-HB, NEFA, AST, and blood urea nitrogen (BUN). Dystocia showed only weak early-window signals (A:G ratio, NEFA), consistent with its small event count (*n* = 11).

**Figure 2:**
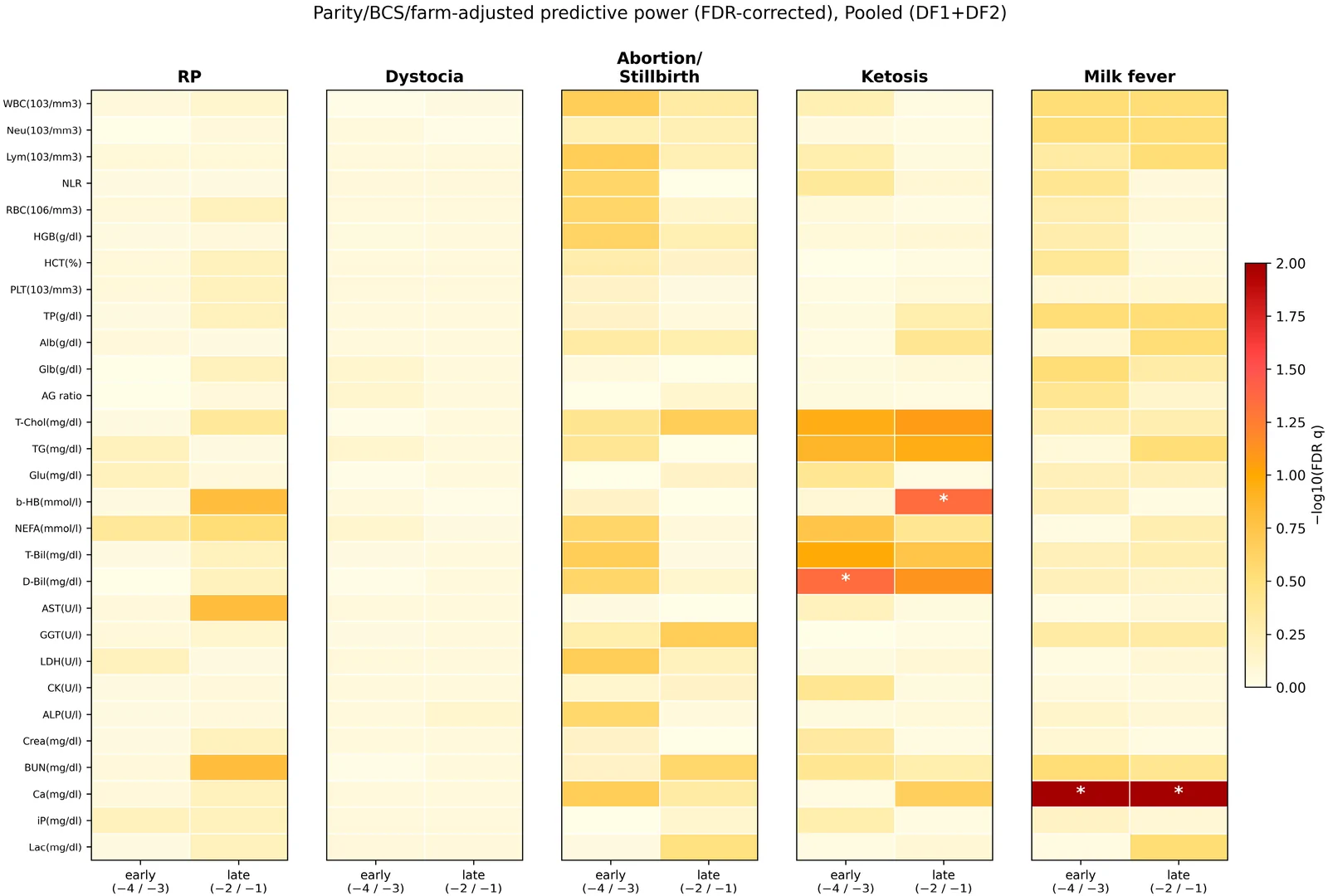
Disease-associated parameters surviving FDR correction (pooled, parity/BCS/farm-adjusted). Benjamini–Hochberg correction was applied within each disease across its 58 tests (2 windows × 29 parameters). Color encodes − log_10_ (FDR *q*); asterisks (*) mark *q* < 0.05. Only calcium for milk fever (both windows) and the bilirubin–ketone markers for ketosis remained significant.

After FDR correction (Figure 2) and after adjustment for parity, BCS, and farm, the markers that remained selected were calcium for milk fever (both windows) and the bilirubin–ketone markers for ketosis (D-Bil in the early window, b-HB in the late window). Ca and D-Bil are therefore the two parameters selected as confounder-robust, disease-associated prepartum markers, and constitute the primary finding of this screening study.

RP illustrates the opposite outcome and explains its exclusion from the selected set. The late-window parameters (higher NEFA and bilirubin, T-Bil and D-Bil, with higher BUN) were significant at the uncorrected level in both the unadjusted and the parity/BCS/farm-adjusted screens, but no RP parameter survived FDR correction in any screen (minimum adjusted *q* = 0.159). In the multivariable model, prepartum NEFA remained associated with RP (OR 1.40 per SD, *p* = 0.013), but overall discrimination was poor (AUC 0.63, cross-validated 0.58). No prepartum parameter for RP, therefore, passed FDR correction.

The unadjusted Welch’s t/Mann–Whitney U screen gave concordant results (Supplementary Figure S3). The principal signals were also present in each farm analyzed separately (Supplementary Figures S1 and S2): calcium (milk fever) and direct bilirubin (ketosis) showed a consistent direction of effect in both DF1 and DF2, indicating that the selection was not driven by a single farm.

### 3.3. Disease-Specific Prepartum Predictor Profiles

**Figure 3:**
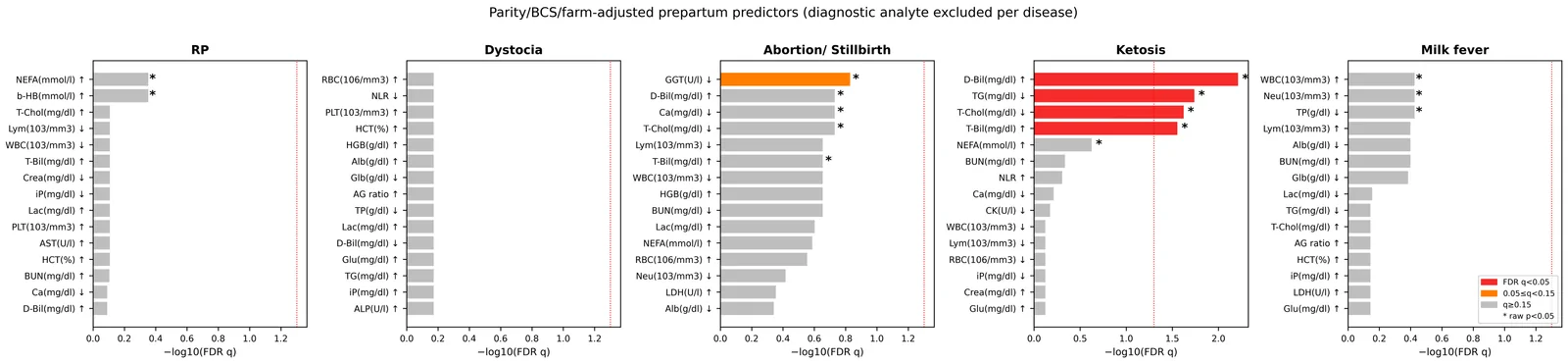
Ranking of non-diagnostic prepartum predictors for each disease (parity/BCS/farm-adjusted). Prepartum parameters (mean of weeks −4 to −1) ranked by strength of association with each disease, excluding each disease’s defining parameter (Ca for milk fever, b-HB for ketosis). Bar length is −log_10_ (FDR *q*); color indicates significance (red, *q* < 0.05; orange, 0.05 ≤ *q* < 0.15; gray, *q* ≥ 0.15) and the arrow indicates direction (↑ higher, ↓ lower in affected calvings). The dotted red line marks the FDR threshold of *q* = 0.05. Asterisks (*) mark raw *p* < 0.05.

To identify predictors that are not simply restatements of the diagnosis, each disease’s defining parameter was excluded (Ca for milk fever, b-HB for ketosis) and the remaining prepartum parameters (mean of weeks −4 to −1) were ranked by their adjusted association with each disease (Figure 3). Ketosis retained four non-diagnostic predictors surviving FDR correction—higher direct and total bilirubin (D-Bil, T-Bil) with lower total cholesterol (T-Chol) and triglycerides (TG), all at *q* < 0.05. No non-diagnostic parameter reached *q* < 0.05 for the other four outcomes; the strongest was GGT for abortion/stillbirth (0.05 ≤ *q* < 0.15). Prepartum NEFA was associated with ketosis at the uncorrected level (adjusted *p* = 0.042, higher in affected calvings) but did not survive FDR correction, and is flagged as raw-significant in Figure 3.

Two-parameter median-split combinations were examined only as an exploratory analysis (Supplementary Figure S4). Given the small number of events for several outcomes and the large number of possible parameter pairs, these combinations were not treated as confirmatory and are not interpreted further here.

### 3.4. Multivariable Prediction of Disease from Prepartum Parameters

Pooled multivariable logistic models were fitted for the three outcomes with adequate events (RP, ketosis, milk fever; Table 2); dystocia and abortion/stillbirth were not modeled (events < 20). Milk fever was the best-discriminated outcome (*n* = 400, 66 events; AUC = 0.81), driven by lower Ca and higher BUN. Ketosis was discriminated (*n* = 400, 86 events; AUC = 0.75) by lower TG and T-Chol and higher BUN. RP was only weakly discriminated (*n* = 399, 53 events; AUC = 0.63); prepartum NEFA was associated with RP (OR = 1.40 per SD, *p* = 0.013), but overall discrimination was poor. Farm (DF1) was non-significant in every model. On internal five-fold cross-validation, AUCs were close to the in-sample values (milk fever 0.78, ketosis 0.71, RP 0.59), preserving the same ranking.

Accounting for repeated calvings by animal (cluster-robust generalized estimating equations, GEEs) left the odds ratios essentially unchanged: milk fever–Ca 0.32→0.31, ketosis–TG 0.69→0.69, –T-Chol 0.68→0.67, –BUN 1.55→1.57; RP–NEFA 1.40→1.39 (Supplementary Table S1). On cross-farm validation (training on one farm and testing on the other), AUCs were 0.74–0.86 for milk fever, 0.69–0.78 for ketosis, and 0.61–0.62 for RP (Supplementary Table S2).

## 4. Discussion

### 4.1. Prepartum Markers of Milk Fever and Ketosis

This study identified a small set of prepartum parameters, obtainable from a standard biochemistry panel, that were associated with subsequent milk fever and ketosis. Screening 29 prepartum parameters against five periparturient diseases, with adjustment for parity, BCS, and farm, and correction for multiple testing, selected a deliberately compact marker set. Two markers were robust: low prepartum calcium for milk fever and a lipid–bilirubin profile for ketosis. For milk fever, the predictor is prepartum calcium itself, reflecting the subclinical disruption of calcium homeostasis that becomes clinical after calving; an independent, distinct risk associated with higher parity is consistent with the known age-related decline in calcium-mobilization capacity [18]. Notably, this signal was already identifiable during the early prepartum phase, approximately three to four weeks before calving, and sustained through the late prepartum period—earlier than the single close-to-calving sampling used in most prior work [18,19]—extending the practical window for early identification. For ketosis, the parity/BCS-adjusted signature was lower total cholesterol and triglycerides with rising bilirubin and NEFA, reproducing the established negative-energy-balance phenotype in which adipose mobilization elevates NEFA and, through incomplete hepatic oxidation, b-HB, while hepatic lipid infiltration and lipoprotein consumption lower circulating cholesterol [13,16,17,27]. The convergence of the multivariable ketosis model with this pathway indicates that the selected markers are biologically coherent rather than statistical artifacts. Two further checks addressed their statistical robustness. Because some cows contributed more than one calving, the models were re-estimated with clustering by animal; the odds ratios were essentially unchanged, showing that the non-independence of repeated calvings did not affect the conclusions. In cross-farm validation, discrimination was retained when a model trained on one farm was applied to the other for milk fever and ketosis, suggesting that these associations are not confined to a single herd, although this falls short of external validation.

### 4.2. Signals for Retained Placenta and Their Robustness

A bilirubin–RP association was reported through untargeted plasma metabolomics [21,22,28], whereas classical RP predictors emphasize NEFA, BCS, b-HB, AST, and GGT [20]. Using only a routine clinical biochemistry panel, we reproduced a prepartum bilirubin signal for RP, indicating it is detectable without mass-spectrometry platforms. However, unlike the ketosis–bilirubin association—which survived adjustment, FDR correction, and separate-farm analysis—the RP–bilirubin signal did not survive adjustment and was confined to the uncorrected screen; a late-phase increase in NEFA was likewise only weakly associated in the multivariable model (OR 1.40 per SD, *p* = 0.013) against poor overall discrimination (AUC 0.63). Both are therefore best regarded as hypothesis-generating rather than confirmed predictors. Safak et al. [29] reported elevated GGT, AST, and bilirubin in Simmental cows with retained fetal membranes, mastitis, or metritis, but these samples were collected within 21 days after calving—that is, after disease had occurred. In our prepartum data, the same hepatic parameters were associated with RP only at the uncorrected level, suggesting that hepatic markers of RP become detectable most clearly once disease is established. A shared mechanism could nonetheless underlie the prepartum bilirubin and NEFA signals: when prepartum fat mobilization raises NEFA beyond the liver’s oxidative capacity, triglycerides accumulate in hepatocytes (subclinical fatty liver), and the resulting reduction in bilirubin conjugation and biliary excretion raises circulating bilirubin, so a prepartum rise in bilirubin may mark hepatocellular stress already present before calving [27,30,31]. Because hepatic dysfunction and oxidative stress impair neutrophil chemotaxis and phagocytosis, and because neutrophil infiltration of the caruncle–cotyledon interface is required for normal placental separation, impaired neutrophil competence offers a candidate route from prepartum hepatic stress to RP [32]. Prepartum bilirubin may thus be a shared readout of subclinical hepatic–oxidative compromise rather than a specific marker of either disease. This interpretation is consistent with the performance of the RP model itself: cross-farm discrimination remained low, mirroring the limited discrimination already observed in the pooled model and reinforcing our view that the RP signal is hypothesis-generating rather than a usable predictor.

### 4.3. Practical Implications: Sampling Timing, Disease Onset, and Parity

Because both milk fever and ketosis markers were detectable in the early and late windows, a single prepartum sample timed to routine herd-health visits—rather than a precise gestational day—may capture much of the available predictive signal, which is more feasible for dairy farms than a strict weekly protocol. Prepartum divergence was concentrated in early-onset disease, so prepartum screening preferentially identifies early-onset, metabolically driven cases, which may partly explain the imperfect discrimination of prepartum models [27].

In an exploratory parity-stratified analysis, which reduced each outcome to 22–54 events per subgroup, only the milk fever–calcium association survived FDR correction, and it did so in both primiparous and multiparous cows, indicating a parity-independent calcium signal. For ketosis, no parameter survived correction within either subgroup; at the uncorrected level, the lipid–bilirubin panel (direct and total bilirubin, triglycerides, total cholesterol) was most evident in multiparous cows, whereas a neutrophil-to-lymphocyte signal was more prominent in primiparous cows. These subgroup patterns are underpowered and are reported only as hypothesis-generating. Where screening is applied, the calcium and direct-bilirubin cutoffs achieved negative predictive values above 89% but modest positive predictive values, making them suited to low-cost triage—reliably reassuring cows below the threshold—rather than stand-alone diagnosis.

Although two-parameter combinations were exploratory and no interaction was statistically significant after accounting for the many possible pairs, one combination is of practical note: cows with both high direct bilirubin and low total cholesterol before calving had a ketosis incidence of 32% (23/73), about 2.6-fold that of the remaining cows (12%) and nearly double the overall rate (17%) (Supplementary Figure S4). Jointly abnormal bilirubin and cholesterol may therefore flag a high-risk subgroup more sharply than either parameter alone, a pattern worth testing prospectively.

### 4.4. Limitations

Several limitations should be noted. The first concerns the outcomes themselves: low frequencies of dystocia and abortion/stillbirth (4–14 events per farm) precluded robust multivariable modeling or reliable farm-specific inference; consequently, findings for these conditions should be considered strictly hypothesis-generating, while twinning was excluded entirely. Related to this, although collapsing ketosis and milk fever into binary outcomes preserved statistical power, this approach discards a potentially predictable severity gradient. A second limitation concerns the study design. Because calvings were sampled on two alternating biweekly schedules, the early and late windows each span approximately two weeks; this grouping retained the full analytic sample and reflects sampling as it is actually performed on commercial farms, but it prevents the resolution of predictive signals to a specific gestational week. In addition, because some cows contributed more than one calving, calving events were not fully independent; we therefore confirmed that cluster-robust models accounting for this non-independence yielded essentially unchanged estimates. A third limitation concerns the modeling approach. Forward stepwise selection can be unstable and optimistic, and although we mitigated this through candidate pre-screening, removal of highly correlated candidates, and both internal and cross-farm validation, some residual instability cannot be excluded.

Most importantly, total and direct bilirubin data exhibited systematic, animal-level missingness that varied substantially by farm (34.8% overall missing rate; missingness for each parameter, by window and at the calving-event level, is given in Supplementary Table S3). This pattern is consistent with an unannounced change in the biochemistry panel configuration during data collection. This could bias the estimated effect size in either direction, and the strength of the association should therefore be regarded as provisional. The bilirubin signal for ketosis was, however, directionally consistent when the two farms were examined separately, indicating that it is not an artifact of a single farm’s panel. Moreover, bilirubin was not retained in any final multivariable model, so the models in Table 2 were fitted on complete-case samples of approximately 400 calving events and were not restricted by this missingness. Nevertheless, the bilirubin-based findings should be treated as hypothesis-confirming rather than definitive, and re-evaluation in a cohort with complete bilirubin measurement is a priority for future work.

Finally, because the two study farms are located within a single dairy region, and the strongest two-parameter interactions rest on relatively few events amid numerous potential combinations, the generalizability of these results is constrained. These limitations underscore the need for prospective confirmation in larger, multi-farm cohorts using a single, fixed sampling schedule.

## 5. Conclusions

In this retrospective two-farm study, screening routine prepartum blood chemistry against five periparturient diseases, with adjustment for parity, BCS, and farm and FDR correction, selected a compact set of disease-associated markers. Two were robust after confounder adjustment: low prepartum calcium for milk fever and a lipid–bilirubin profile (elevated direct/total bilirubin with reduced cholesterol and triglycerides) for ketosis, both detectable three to four weeks before calving and confirmed in multivariable models (milk fever: AUC 0.81 in-sample, 0.78 on internal five-fold cross-validation; ketosis: 0.75 and 0.71, respectively), with discrimination retained when models were applied across farms. Obtainable from routine biochemistry, these parameters are candidate markers for further investigation, most applicable to multiparous cows, although the retrospective design and the inclusion of only two farms mean these findings should be regarded as hypothesis-generating for clinical use. A bilirubin–RP signal was detectable but did not survive confounder adjustment and remains hypothesis-generating. Prospective confirmation in larger, multi-farm cohorts with complete parameter panels and a single fixed sampling schedule is warranted before clinical adoption.

## Figures and Tables

**Table 1:** Study population and disease incidence by farm. Summary of the number of animals, calving events, parity, and prepartum body condition score (BCS) for each farm (DF1 and DF2), with the incidence of the five outcomes. The analytic unit is the calving event (animal ID × parity). Incidence is expressed as a percentage of calving events at that farm. Abbreviations: BCS, body condition score; RP, retained placenta.

Farm	DF1	DF2
Cows (*n*)	158	115
Calvings (*n*)	248	171
Parity, mean ± SD	1.74 ± 0.97	2.41 ± 1.45
BCS (prepartum mean), mean ± SD	3.37 ± 0.15	3.36 ± 0.17
Retained Placenta Event *n* (%)	31 (12.5%)	24 (14.0%)
Dystocia Event *n* (%, calving criterion)	5 (2.0%)	6 (3.5%)
Abortion/Stillbirth Event *n* (%, calving criterion)	4 (1.6%)	14 (8.2%)
Ketosis Event *n* (%, calving criterion)	63 (25.4%)	28 (16.4%)
Milk fever Event *n* (%, calving criterion)	32 (12.9%)	37 (21.6%)

**Table 2:** Pooled multivariable logistic models predicting RP, ketosis, and milk fever from prepartum parameters (weeks −4 to −1). Selected parameters with odds ratios (OR, per 1 SD), 95% confidence intervals (CIs), and *p*-values, with model-level sample size (events), in-sample AUC, 5-fold cross-validated AUC, and McFadden’s pseudo-R^2^. All models were adjusted for parity, BCS, and farm (DF1); parameters were selected by forward stepwise AIC. Dystocia and abortion/stillbirth were not modeled (events < 20). Abbreviations: T-Chol, total cholesterol; NEFAs, non-esterified fatty acids; TGs, triglycerides; iP, inorganic phosphorus; BUN, blood urea nitrogen; Ca, calcium; ALP, alkaline phosphatase; OR, odds ratio; CI, confidence interval; AUC, area under the receiver operating characteristic curve; SD, standard deviation; AIC, Akaike information criterion; RP, retained placenta.

Outcome	Parameter	OR (per 1 SD)	95% CI	*p*-Value	*n* (Events)	AUC	CV AUC	Pseudo-R^2^
Retained placenta	T-Chol	1.35	0.98–1.84	0.062	399 (53)	0.63	0.59	0.04
	NEFA	1.40	1.07–1.82	0.013				
Ketosis	TG	0.69	0.50–0.94	0.021	400 (86)	0.75	0.71	0.12
	T-Chol	0.68	0.50–0.92	0.012				
	iP	0.86	0.66–1.11	0.239				
	BUN	1.55	1.16–2.06	0.003				
Milk fever	Ca	0.32	0.23–0.46	<0.001	400 (66)	0.81	0.78	0.22
	BUN	1.41	1.04–1.92	0.028				
	ALP	0.76	0.49–1.18	0.219				

Youden-optimal cutoffs for the two markers were, for milk fever, Ca ≤ 9.08 mg/dL in the early window (sensitivity 69%, specificity 57%; AUC = 0.67) and ≤8.49 mg/dL in the late window (sensitivity 45%, specificity 93%; AUC = 0.73), and for ketosis, D-Bil ≥ 0.051 mg/dL (early) and ≥0.081 mg/dL (late; AUC = 0.67–0.68). Negative predictive value exceeded 89% at every window, whereas positive predictive value was modest (23–58%).

## Data Availability

Data are contained within the article or Supplementary Material.

## Supplementary Materials

The following supporting information can be downloaded at https://www.mdpi.com/article/10.3390/ani16182946/s1. Table S1: Sensitivity analysis for repeated calvings; Table S2: Cross-farm_validation_of_the_multivariable_models; Table S3: Missing data for each blood parameter; Figure S1: Parity/BCS-adjusted predictive-power heatmap by farm (raw p); Figure S2: Parity/BCS-adjusted predictive-power heatmap by farm (FDR-corrected); Figure S3: Unadjusted predictive-power heatmap (pooled, Welch’s t/Mann–Whitney U); Figure S4: Exploratory two-parameter risk stratification.

## Abbreviations

AG ratio	albumin-to-globulin ratio
AIC	Akaike information criterion
Alb	albumin
ALP	alkaline phosphatase
AST	aspartate aminotransferase
AUC	area under the receiver operating characteristic curve
BCS	body condition score
b-HB	β-hydroxybutyrate
BUN	blood urea nitrogen
Ca	calcium
CI	confidence interval
CK	creatine kinase
Crea	creatinine
CV	cross-validation
D-Bil	direct bilirubin
DF1, DF2	dairy farm 1, dairy farm 2
FDR	false discovery rate
GGT	γ-glutamyl transferase
Glb	globulin
Glu	glucose
HCT	hematocrit
HGB	hemoglobin
iP	inorganic phosphorus
Lac	lactate
LDH	lactate dehydrogenase
Lym	lymphocytes
NEFAs	non-esterified fatty acids
Neu	neutrophils
NLR	neutrophil-to-lymphocyte ratio
OR	odds ratio
PLT	platelets
RBCs	red blood cells
RP	retained placenta
SD	standard deviation
T-Bil	total bilirubin
T-Chol	total cholesterol
TGs	triglycerides
TP	total protein
WBCs	white blood cells
