# Supplementary material for: Prepartum Blood Biochemistry and Periparturient Disease Risk in Dairy Cows: A Retrospective Two-Farm Study
Source: Animals (Basel). 2026 Sep 19;16(18):2946. doi: 10.3390/ani16182946 (PMC13603721; doi:10.3390/ani16182946)
Supplement: Supplementary file 1 [file animals-16-02946-s001.zip › SupplementaryTableS1_Sensitivity_analysis_for_repeated_calvings.pdf]

Supplementary Table S1. Sensitivity analysis for repeated calvings: standard logistic regression versus cluster-robust models accounting for within-cow correlation.

**Outcome (n, events)**	**Parameter**	**Standard logistic OR (95% CI)**	***p*-value**	**Cluster-robust (GEE) OR (95% CI)**	***p*-value**
**Retained placenta (399, 53 events)**	T-Chol	1.35 (0.98–1.84)	0.062	1.34 (1.03–1.74)	0.028
	NEFA	1.40 (1.07–1.82)	0.013	1.39 (1.11–1.75)	0.004
**Ketosis (400, 86 events)**	TG	0.69 (0.50–0.94)	0.021	0.69 (0.49–0.96)	0.027
	T-Chol	0.68 (0.50–0.92)	0.012	0.67 (0.49–0.91)	0.010
	iP	0.86 (0.66–1.11)	0.239	0.82 (0.63–1.07)	0.148
	BUN	1.55 (1.16–2.06)	0.003	1.57 (1.18–2.08)	0.002
**Milk fever (400, 66 events)**	Ca	0.32 (0.23–0.46)	< 0.001	0.31 (0.21–0.47)	< 0.001
	BUN	1.41 (1.04–1.92)	0.028	1.41 (1.07–1.86)	0.016
	ALP	0.76 (0.49–1.18)	0.219	0.79 (0.51–1.23)	0.293

Because 97 of 273 cows (36%) contributed more than one calving, each final model was re-estimated using generalized estimating equations (GEE) with an exchangeable working correlation clustered by animal, which accounts for the non-independence of repeated calvings. Both models were adjusted for parity, BCS, and farm, whose coefficients are omitted here. Odds ratios (OR) are per 1 SD increase. CI, confidence interval.
