# Supplementary material for: Prepartum Blood Biochemistry and Periparturient Disease Risk in Dairy Cows: A Retrospective Two-Farm Study
Source: Animals (Basel). 2026 Sep 19;16(18):2946. doi: 10.3390/ani16182946 (PMC13603721; doi:10.3390/ani16182946)
Supplement: Supplementary file 1 [file animals-16-02946-s001.zip › SupplementaryTableS2_Cross-farm_validation_of_the_multivariable_models.pdf]

Supplementary Table S2. Cross-farm validation of the multivariable models.

| Outcome | Training set: n (events) | Test set: n (events) | AUC in test farm |
|---|---|---|---|
| **Retained placenta** | DF1: 237 (30) | DF2: 162 (23) | 0.62 |
| | DF2: 162 (23) | DF1: 237 (30) | 0.61 |
| **Ketosis** | DF1: 239 (60) | DF2: 161 (26) | 0.78 |
| | DF2: 161 (26) | DF1: 239 (60) | 0.69 |
| **Milk fever** | DF1: 240 (32) | DF2: 160 (34) | 0.86 |
| | DF2: 160 (34) | DF1: 240 (32) | 0.74 |

Each model was fitted on one farm only (parity, BCS, and the selected parameters; the farm term was omitted because it is constant within a farm) and applied without refitting to the other farm, so that no information from the test farm entered model estimation. Selected parameters were those of the pooled models (Table 2). AUC, area under the receiver operating characteristic curve; DF1, DF2, dairy farms 1 and 2.
