# Supplementary material for: Prepartum Blood Biochemistry and Periparturient Disease Risk in Dairy Cows: A Retrospective Two-Farm Study
Source: Animals (Basel). 2026 Sep 19;16(18):2946. doi: 10.3390/ani16182946 (PMC13603721; doi:10.3390/ani16182946)
Supplement: Supplementary file 1 [file animals-16-02946-s001.zip › SupplementaryTableS3_Missing_data_for_each_blood_parameter.pdf]

Supplementary Table S3. Missing data for each blood parameter (n = 419 calving events).

| Parameter | Early window (week −4/−3) n (%) | Late window (week −2/−1) n (%) | Calving event (any prepartum sample) n (%) |
|---|---|---|---|
| Lactate (Lac) | 212 (50.6%) | 197 (47.0%) | 175 (41.8%) |
| Direct bilirubin (D-Bil) | 184 (43.9%) | 174 (41.5%) | 146 (34.8%) |
| Total bilirubin (T-Bil) | 184 (43.9%) | 174 (41.5%) | 146 (34.8%) |
| Aspartate aminotransferase (AST) | 93 (22.2%) | 94 (22.4%) | 48 (11.5%) |
| γ-glutamyl transferase (GGT) | 90 (21.5%) | 83 (19.8%) | 41 (9.8%) |
| Creatine kinase (CK) | 68 (16.2%) | 72 (17.2%) | 19 (4.5%) |
| β-hydroxybutyrate (b-HB) | 71 (16.9%) | 56 (13.4%) | 9 (2.1%) |
| Non-esterified fatty acids (NEFA) | 65 (15.5%) | 57 (13.6%) | 7 (1.7%) |
| Creatinine (Crea) | 55 (13.1%) | 44 (10.5%) | 5 (1.2%) |
| Lactate dehydrogenase (LDH) | 57 (13.6%) | 44 (10.5%) | 5 (1.2%) |
| Alkaline phosphatase (ALP) | 59 (14.1%) | 44 (10.5%) | 5 (1.2%) |
| Blood urea nitrogen (BUN) | 55 (13.1%) | 44 (10.5%) | 5 (1.2%) |
| Platelets (PLT) | 51 (12.2%) | 44 (10.5%) | 4 (1.0%) |
| Inorganic phosphorus (iP) | 55 (13.1%) | 43 (10.3%) | 4 (1.0%) |
| Albumin-to-globulin ratio (A:G) | 54 (12.9%) | 42 (10.0%) | 3 (0.7%) |
| Globulin (Glb) | 54 (12.9%) | 42 (10.0%) | 3 (0.7%) |
| Albumin (Alb) | 54 (12.9%) | 42 (10.0%) | 3 (0.7%) |
| Total cholesterol (T-Chol) | 55 (13.1%) | 42 (10.0%) | 3 (0.7%) |
| Hematocrit (HCT) | 51 (12.2%) | 41 (9.8%) | 3 (0.7%) |
| Hemoglobin (HGB) | 51 (12.2%) | 42 (10.0%) | 3 (0.7%) |
| Red blood cells (RBC) | 51 (12.2%) | 41 (9.8%) | 3 (0.7%) |
| White blood cells (WBC) | 51 (12.2%) | 41 (9.8%) | 3 (0.7%) |
| Neutrophil-to-lymphocyte ratio (NLR) | 51 (12.2%) | 41 (9.8%) | 3 (0.7%) |
| Lymphocytes (Lym) | 51 (12.2%) | 41 (9.8%) | 3 (0.7%) |
| Neutrophils (Neu) | 51 (12.2%) | 41 (9.8%) | 3 (0.7%) |
| Total protein (TP) | 54 (12.9%) | 42 (10.0%) | 3 (0.7%) |
| Triglycerides (TG) | 54 (12.9%) | 42 (10.0%) | 3 (0.7%) |
| Glucose (Glu) | 54 (12.9%) | 42 (10.0%) | 3 (0.7%) |
| Calcium (Ca) | 55 (13.1%) | 43 (10.3%) | 3 (0.7%) |

Values are the number (%) of calving events without a measurement. The early and late window columns refer to the single prepartum sample assigned to each window; the final column indicates calving events with no prepartum measurement of that parameter in either window. Missingness is therefore lower at the calving-event level, because a calving event remains analyzable when only one of its two prepartum samples was measured. Parameters are ordered by calving-event missingness.
